# 558 Wound Care Program Development to Advance Physical Therapy Practice in a Specialized Burn Unit

**DOI:** 10.1093/jbcr/irad045.154

**Published:** 2023-05-15

**Authors:** Nicole Gerlich, Melinda Tepe

**Affiliations:** VCU Health System, Henrico, Virginia; VCU Health System, Mechanicsville, Virginia; VCU Health System, Henrico, Virginia; VCU Health System, Mechanicsville, Virginia

## Abstract

**Introduction:**

There is a nation-wide shortage of nurses which is true at our institution as well. As a result, there is a lack of consistent, qualified staff to perform wound care in our burn center. Wound care is within a physical therapist’s (PT) scope of practice and we felt this was an area for opportunity and expansion for PT practice at our institution as we had not previously been involved in wound care services for any patient population.

**Methods:**

We posted on the ABA PT/OT SIG page asking other burn centers about PT involvement in daily wound care. Respondents then participated in an online question/answer session. Based on this information, additional education resources, and our own institution’s practice model for nursing, we developed a PT checklist for demonstrating competency with theory and practice for burn wound care. The 2 primary burn PTs completed a 4 week training under the direction of the nurse clinical coordinators and advanced practice providers (APPs) and were deemed competent. The attending MDs have signed off on our process. We have now developed a training program to ensure more PTs are competent with wound care for the burn patient.

**Results:**

Of 186 members of the OT/PT SIG who had access to the original post, we received only 8 responses. Five of eight had PTs partially involved in wound care in the inpatient setting with burn patients. PTs are the primary wound care providers in 2 of 8 responding burn centers. In the remainder, PTs were primarily involved in wound care only in the outpatient setting. Four centers participated in the online video question and answer sessions. Despite all of them following ABA guidelines, there was no standardization of developing a wound care program. Our program includes 1:1 orientation to the burn unit for 3-4 weeks, direct supervision of wound care and dressing changes by the primary burn PTs, completion of knowledge-based quiz, and demonstration of skills checklist.

**Conclusions:**

It was feasible to create a new wound care program including PTs in a burn center. We found variation in practice due to regional preferences, physician preferences, availability of different dressings, and staff training. We feel confident in our competency process as it includes both a written knowledge assessment as well as requiring demonstration of techniques. Developing this program has increased the trust between the PTs, physicians, and APPs thereby allowing us to advance our PT practice. We can now perform high quality wound care and complete more aggressive functional mobility without fear of disrupting dressings. Based on actual visualization of the wounds, we can provide more thorough patient education and perform more specific range of motion stretching.

**Applicability of Research to Practice:**

Utilizing PTs knowledge of all soft tissue healing may be of benefit. We encourage other PT departments to advocate to participate in wound care of the burn patient.

